# Approach to Anemia in Hospitalized Patients with Infectious Diseases; Is it Appropriate?

**Published:** 2015

**Authors:** Taher Entezari-Maleki, Hossein Khalili, Iman Karimzadeh, Sirous Jafari

**Affiliations:** a *Department of Clinical Pharmacy, Faculty of Pharmacy, Tabriz University of Medical Sciences, Tabriz, Iran.*; b *Department of Clinical Pharmacy, Faculty of Pharmacy, Tehran University of Medical Sciences, Tehran, Iran.*; c *Department of Clinical Pharmacy, Faculty of Pharmacy, Shiraz University of Medical Sciences, Shiraz, Iran. *; d *Department of Infectious Diseases, Imam Khomeini Hospital Complex, Faculty of Medicine, Tehran University of Medical Sciences, Tehran, Iran.*

**Keywords:** Anemia of chronic disease, Prevalence, Infectious diseases, Mortality

## Abstract

Anemia of chronic diseases (ACD) is a common problem in patients with infectious diseases and can influence the quality of life and patients' survival. Despite the clinical importance of ACD, data are still lacking regarding this problem in the infectious diseases. This study aimed to evaluate the prevalence, related factors, outcome and approaches to anemia in the infectious diseases ward.

This retrospective study was performed to review the medical records of patients admitted to the infectious diseases department of Imam Khomeini hospital during a two-year period between 2009 and 2011. A standard protocol was developed to evaluate anemia. Patients’ demographic data approaches to manage anemia and routine laboratory tests were recorded and compared with the protocol.

Totally, 1,120 medical records were reviewed. ACD was recognized in 705 patients (63%). Only 5.1% of diagnostic and 8.7% of treatment approaches was based on the protocol. The majority of patients (89.4%) were received inappropriate treatment regarding. Mortality rate of patients with ACD was 3.4%. Moreover, a significant correlation between anemia and mortality was detected (r = 0.131; p = 0.026). A statistically significant correlation was also identified between patients’ Hgb and ESR, CRP, reasons of admission, number of medications, and underlying diseases.

In conclusion, results of this study suggested that ACD is a common problem in infectious diseases patients and significantly associated with patients’ mortality. Moreover, the majority of studied patients were not received an appropriate diagnostic and treatment approach which arises more concerns regarding the management of ACD in infectious diseases setting.

## Introduction

Anemia of chronic diseases (CAD) is the most common type of anemia after iron deficiency type and generally involves patients with chronic diseases. Immune system plays the main role in the pathophysiology of ACD that lead to dysregulation of iron homeostasis. In this condition, despite normal or high iron body stores, blood level of iron generally remains low (1-5).

A number of chronic diseases such as infectious and inflammatory diseases, kidney dysfunction, and cancers can cause ACD. Among these, acute and chronic infectious diseases are the most common causes with reported prevalence of 18-95% (1, 6-8). The main infectious diseases that cause ACD include tuberculosis, human immunodeficiency virus (HIV), endocarditis, and osteomyelitis. However, acute infections also may cause ACD. Anemia associated with HIV infection can reduce patients’ survival and quality of life (QOL) and is an independent predictor for clinical outcome (9-13). Therefore, proper approach to anemia in chronic diseases is one of the important parts of individual management that can improve quality of care and survival of patients. Despite the importance of this issue, data are still lacking regarding anemia in subjects admitted to infectious diseases wards. Therefore, this study aimed to evaluate the prevalence, related factors, outcome, and approaches to anemia in an infectious diseases ward. 

## Experimental

*Study design and setting*

We designed a retrospective cohort study to review the medical records of all patients admitted from 2009 to 2011 to the infectious diseases wards of Imam Khomeini Hospital Complex, a tertiary, teaching setting affiliated to Tehran University of Medical Sciences, Tehran, Iran. The institutional review board and the Medical Ethics Committee of the hospital approved this study. 

*Protocol design*

After literature review, a study protocol was developed for the evaluation of anemia (1,14-15). According to this protocol, all males and females with the hemoglobin (Hgb) levels less than 13 g/dL and 12 g/dL respectively, met the criteria of anemia, were entered into the study.

The diagnostic approach of anemia was as follows: patients with anemia and biochemical or clinical evidences of inflammation with transferrin saturation (T-sat) of <16% were divided into three groups based on the ferritin levels. Patients with ferritin levels of <30 ng/mL and > 100 ng/mL were considered as iron deficiency anemia and ACD, respectively. Those with ferritin of 30-100 ng/mL and the ratio of concentration of soluble transferrin receptor to log of the serum ferritin level (sTfR/log ferritin) < 1 were also determined as ACD. Individuals with ferritin of 30-100 ng/mL and sTfR/log ferritin > 2 were classified as ACD with true iron deficiency.

Based on the protocol, treatment approach to ACD was blood transfusion in the context of either severe (hemoglobin less than 8.0 g/dL) or life-threatening (hemoglobin less than 6.5 g/dL) anemia. Iron supplementation was indicated in patients with ACD concomitant with absolute iron deficiency. It was also considered for those with functional iron deficiency unresponsive to erythropoietic agents. However, iron therapy was not taken into account for patients with ACD who have ferritin level of > 100 ng/mL due to possible adverse outcomes in this setting.

Erythropoietic agents as drugs of choice for ACD management (1-5) were initiated for patients with hemoglobin levels ranged between 11 and 12 g/dL. In accordance to the protocol, monitoring of therapy with erythropoietic agents was checking hemoglobin levels after four weeks of starting the therapy and at intervals of two to four weeks thereafter (1).

*Data collection*

Patients’ demographic data (sex, age, and weight), past medical, habitual, family and social histories, medications and allergies, present illness, diagnostic and treatment approaches to manage anemia, and routine lab tests including complete blood counts (CBC), platelet, erythrocyte sedimentation rate (ESR), C-reactive protein (CRP), serum iron, ferritin, and transferin levels were recorded. 

Diagnostic and treatment approaches were compared with the protocol and categorized as appropriate, partially appropriate, or inappropriate.

*Data Analysis*

Data analyses were performed by the SPSS version 16 software. Kolmogorov-Smirnov test was conducted to assess normal distribution of data. Wilcoxon, Spearman, and Chi-square tests were used to evaluate the relationship between anemia and studied factors. p-values less than 0.05 were considered statistically significant.

## Results

During the study period, medical records of 1,120 patients were reviewed. ACD was detected in 63% (n=705) of patients included 384 (54.5%) males and 321 (45.5%) females. The mean ± SD for age and weight of participants was 47 ± 18.5 years and 62.5 ± 15 Kg, respectively. Soft tissue infections 232 (33%), tuberculosis 90 (12.8%), and endocarditis 56 (7.9%) were the most common causes of ward admission (Table 1). Diabetes mellitus and cardiovascular diseases were the most common underlying illnesses (Table 2). 

**Table 1:** Causes of patients hospital admission*.

**Disease**	**Frequency**	**Percent**
Soft tissue infection	232	33
Tuberculosis	90	12.8
Endocarditis	56	7.9
Osteomyelitis	51	7.2
AIDS	28	4
Pneumonia	27	3.8
Urinary tract infection	26	3.7
Septic arthritis	23	3.3
Pyelonephritis	19	2.7
Fever unknown origin	18	2.6
Brucellosis	16	2.3
Meningitis	11	1.6
Diabetic foot	7	1.0
Mucormycosis infection	6	0.9
Hepatitis C	6	0.9
Hepatitis B	2	0.3
Other infections	87	12.1
Total	705	100

* Causes of the patients’ hospital admission have been summarized in this table. Soft tissue infections followed by tuberculosis and endocarditis were the most common causes of hospital admission in the patients.

**Table 2:** Baseline diseases of patients*

**Disease**	**Frequency**	**Percent**
Cardiovascular diseases	179	24.4
Diabetes mellitus	178	24.3
Malignancy	48	6.9
HIV infection	47	6.7
Injection drug user	38	5.4
Hepatitis C	32	4.5
HIV+ Hepatitis C	26	3.7
Tuberculosis	22	3.7
Brucellosis	22	3.1
Major surgery	22	3.1
Chronic kidney diseases	21	3
Hepatitis B	20	2.8
Others	34	4.8

* Baseline diseases of the patients were summarized in this table. Cardiovascular diseases and diabetes mellitus are the most frequent diseases in the patients.

The mean ± SD number of medications received per patient was 9.3 ± 5.5. Anti-diabetic and cardiovascular agents were the most common administered drugs (Table 3). 

The mortality rate of patients with ACD was 3.4% (n=24). A statistically significant correlation between anemia and mortality was detected (r = 0.131, p = 0.026). 

**Table 3:** Past drug history of the patients*.

**Drugs**	**Frequency**	**Percent**
Cardiovascular agents	163	22.9
Anti-Diabetes agents	158	22.6
Opioids	123	17.6
Anti-HIV agents	76	11
Chemotherapy agents	35	4.9
Antibiotics	32	4.7
Immunosuppressant	30	4.3
Anti-TB agents	18	2.6
Psychiatrics agents	17	2.5
Neurologic agents	15	2.2
Interferon + Ribavirin	13	1.7
Others	16	2.3

* Drug history of the patients has been shown in the table. Most of the patients had history of cardiovascular and anti-diabetic agents’ intake at the time of inclusion.

Anemia parameters of patients were shown in Table 4. According to Wilcoxon test, the mean ± SD CRP level increased significantly from 51.7 ± 3.7 at baseline to 60.7 ± 4.2 during hospitalization course (P=0.014). However, these changes for Hgb and ESR were not significant (Table 5).

A significant correlation was identified between patients’ Hgb and ESR (r = -0.276; p=0.001), CRP (r = -0.157; p=0.002), reasons of admission (r = 0.117; p= 0.003), number of medications (r = -0.109; p=0.001), and underlying diseases (r = -0.152; p= 0.001). 

**Table 4:** Anemia -related laboratory parameters of patients*

**Parameters**	**N**	**Mean**	**Standard deviation**
B12 level	5	542.2	833.65
Transferrin saturation	7	5.3%	386.7
Ferritin	57	114	299.15
Mean corpuscular volume	107	86.3	8.1
Transferrin	125	66.6	64.5
Iron Level	33	60.7	42.2

* The patient’s anemia-related laboratory parameters were summarized in this table. Most of the patients did not have the required laboratory parameters for evaluation of anemia types.

**Table 5:** The patients’ CRP, ESR and Hgb before and after anemia approaches*.

**Parameters**	**Mean ± SD (before)**	**Mean ± SD (after)**	**p-value**
			
Hgb(g/dL)	10.2 ± 5	9.8 ± 1.5	P>0.05
CRP (mg/l)^1^	51.3 ± 37	60.7 ± 42.2	0.014
ESR (mm/h)^2^	75.8 ± 40.5	72 ± 40.5	P>0.05

1 : C-Reactive Protein

2 : Erythrocyte Sedimentation Rate

* The patients’ chronic diseases and anemia parameters before and after anemia approaches have been shown in this table.

Only 5.1% of diagnostic and 8.7% of treatment approaches was in accordance with the protocol. The treatment approach to anemia was appropriate in 7.1%, partially appropriate in 3%, and inappropriate in 89.4% of the study cohort. The diagnostic and treatment approaches for management of anemia are shown in Table 6.

**Table 6:** Diagnostic and treatment approaches to the anemia*.

**Approaches**	**Frequency**	**Percent**
**Diagnostic approaches**	33	4.7
Check of iron level	57	8.1
Check of ferritin level	125	17.7
Check of transsferin level	5	0.7
Check of vitamin B12 level	7	1.6
Check of Transferrin saturation	33	4.7
**Treatment approaches**		
Administration of packed cells	24	3.4
Administration of ferrous sulfate	41	5.8
Administration of folic acid	83	11.8
Administration of vitamin B 12	13	1.8
Administration of epoitin alpha	32	4.5
Administration of supplement	76	10.8
Administration of multivitamin preparations	46	6.5

* Diagnostic approaches for the patients’ anemia have been shown in this table. Approach to anemia of most patients was categorized as inappropriate. In few percent of the patients, appropriate approach was implicated.

## Discussion

Anemia in the infectious diseases setting plays an important role in the treatment strategies and can influence patients’ outcome. Iron overload in this setting can deteriorate patient condition and stimulate growth of pathogenic microorganisms (14). Another important issue in the management of anemia and iron supplementation is association of anemia with the patients’ morbidity and mortality (9-13). Therefore, the appropriate approach and evaluation of iron status in patients with infection is an emergent issue. This study was conducted to evaluate the rate, diagnosis, and management approaches of anemia at a referral infectious diseases ward in Iran. 

According to findings of the present study, prevalence of anemia in patients with infectious diseases was 63% that is consistent to the other reports with the range of 18-95% (1, 6-8). High frequency of anemia in this setting may be due to disease pathophysiology, inflammation, malnutrition, and socioeconomic conditions of these patients. 

One of the main findings of this study was the association between patients’ Hgb levels and mortality in agreement with other studies that showed increased mortality in anemic patients with chronic diseases (11-18). These results highlight the importance of appropriate diagnosis and management of anemia in patients with infectious diseases to improve treatment outcome and patients’ survival and quality of life. 

The mean CRP levels were significantly raised during the admission period and had significant correlation with the Hgb levels. Furthermore, high CRP level is associated with low level of Hgb in the context of chronic inflammatory diseases. It may be due to the influence of immune system activation and inflammation on the acute phase reactant proteins, which play a major role in the pathogenesis of ACD (15, 16). Therefore CRP level can be the predictor of anemia in this setting. 

We also identified a significant correlation between Hgb levels and diagnosis, underlying diseases, and numbers of administered medications. These results suggest that type of underlying diseases, type, and the number of given drugs can influence anemia status. Infectious diseases such as HIV, hepatitis C, TB and their medications such as zidovudine, interferon, rifampin, and ribavirin can result in anemia. Among infectious diseases, HIV, hepatitis C, and TB are the most common underlying diseases that can cause anemia. This finding is in line with previous reports (6, 9, 16-21). Diagnostic and treatment approaches in most of our cases were not according to the standard protocol. Due to the importance of anemia in this setting, more considerations for detection and treatment of anemia are needed. 

This investigation was a retrospective cohort study. We did not follow patients after hospital discharge. Required data for evaluation of anemia had not been recorded in medical records of some patients. Serum levels of hepcidin and other inflammatory cytokines have not been assessed. However, these predictors of anemia and inflammations were not currently common in medical workups at internal wards.

In conclusion, the result of this study demonstrated that anemia of chronic diseases is a common problem in patients with infectious diseases and is associated with patients’ mortality and treatment outcome. Moreover, the majority of patients are not received an appropriate diagnostic and treatment approach for anemia which arises more concerns regarding the management of this condition in infectious diseases. For more evaluation of anemia in the context of infectious diseases, well-designed studies with large sample size are warranted.
